# Cannabis Use and Associated Harms among Schizophrenia Patients in a Nigerian Clinical Setting: A Case–Control Study

**DOI:** 10.3389/fpsyt.2016.00136

**Published:** 2016-08-03

**Authors:** Victor Lasebikan, Olaolu O. Aremu

**Affiliations:** ^1^Department of Psychiatry, College of Medicine, University of Ibadan, Ibadan, Nigeria; ^2^Psychiatric Unit, Ring Road State Hospital, Ibadan, Nigeria

**Keywords:** cannabis, screening and brief intervention, schizophrenia, health risks

## Abstract

**Aim:**

The overall aim of this study was to determine the prevalence of cannabis use among patients with schizophrenia with associated levels of harm in a Nigerian clinical setting.

**Method:**

In this case–control study, consecutive 150 patients with schizophrenia were matched by age and gender with an equal number of patients that utilized the general outpatient department of the State Hospital, Ring Road Ibadan. The alcohol, smoking and substance involvement screening test (ASSIST) was used to obtain prevalence of cannabis use and level of health risk as determined by the ASSIST score. The positive and negative syndrome scale was used to determine the severity of psychosis.

**Results:**

Prevalence of cannabis use among the cases and control group was 10.0 and 2.7%, respectively, *p* = 0.03. Mean ASSIST score was significantly higher among the cases compared with the control, *p* < 0.001. Respondents of male gender and those who were not married were significantly more likely to be cannabis users among patients with schizophrenia (*p* < 0.001 and *p* < 0.02), respectively.

**Conclusion:**

Cannabis use was prevalent among patients with schizophrenia and was associated with health risks. Thus, routine screening for cannabis use and brief intervention is suggested to be integrated into care for adolescents and adults with schizophrenia.

## Introduction

A substantial body of evidence from developed countries of the world indicates an association between adolescent cannabis use and schizophrenia (1–3). Cannabis users have also been found to be three times more likely to develop schizophrenia (4). Research findings from Nigeria (5, 6) corroborate those from the Western countries that mental disorders are highly prevalent among cannabis users and account for the majority of drug-related admissions in Nigerian psychiatric care facilities (7).

There are several plausible mechanisms for this association. For example, administration of very high doses of tetrahydrocannabinol to healthy individuals can produce transient psychotic-like symptoms (2). Thus, it is either that cannabis is a risk factor for the development of schizophrenia or its use in schizophrenia may be to self-medicate the negative symptoms of schizophrenia, since conventional antipsychotics do not treat negative symptoms (8). This is because cannabis stimulates dopamine release (9), the level of which is low in negative symptoms. Consistent with this is the reported higher rates of cannabis use in people with schizophrenia than in the general population (10). Nevertheless, prospective and retrospective studies indicate that the onset of cannabis use typically precedes that of schizophrenia (11), somehow revoking the self-medication hypothesis.

Other models that have emerged in the past decades include genetic influences and early environmental hazards, leading to neurodevelopmental problems (12, 13). Furthermore, the expression of the cannabis-1 (CB-1) receptor reaches a peak around the onset of adolescence, implying that the effects of cannabis on the brain are heightened during this period (14), which is a period just before the peak age of onset of schizophrenia.

Although cannabis use among healthy users may be associated with cognitive impairments (15), structural brain abnormalities, and subthreshold psychotic symptoms (16), cannabis has been found to have some beneficial effects on cognition in a sub-population of patients with schizophrenia (17), thereby having implications for its continued use.

The occurrence of cannabis use within schizophrenia poses a significant burden on patients in terms of outcome (18) and cost of treatment. Considering the health indices of Nigeria, where access to formal mental health care is poor (19), cannabis use in schizophrenia is expected to be associated with poorer outcomes, a higher level of disability, and poorer quality of life. It is, therefore, important to derive data on the correlates of cannabis use among patients with schizophrenia in this environment, the findings of which may be applicable to the development of an intervention program for this population.

The overall aim of this study was to determine the prevalence of cannabis use among patients with schizophrenia in the psychiatric unit of the State Hospital, Ring Road, Ibadan compared with patients attending the general outpatient department (GOPD) of the same hospital, for simple ailments. The sociodemographic correlates of cannabis use, the association between symptom profile of schizophrenia, and the level of cannabis-induced health risk was also sought. It was hypothesized that cannabis use would be more significantly prevalent among patients with schizophrenia compared with the control.

## Methodology

The study was carried out in the psychiatric unit and the GOPD of the State Specialists Hospital, Ring Road, Ibadan, the capital city of Oyo State. The city is located in the South-Western part of the country and is the oldest state capital in Nigeria.

The GOPD is the first port of call for all patients. Since patients do not require referral to be attended to at this point, it is a walk-in clinic, making it a *de facto* primary care center. At the GOPD, the Medical Officer on duty first attends to all patients and sorts them to the various clinics, including the psychiatric clinic.

### Study Design

This was a case–control brief intervention study and the findings reported herein are the results of our baseline assessment. The outcome of the brief intervention will be subsequently published. The cases were patients with schizophrenia while the control group was patients with simple ailments that attended the GOPD of the same study center for simple ailments during the study period. Cases were selected by a multistage sampling method.

In the preliminary stage of the study, the sample frame was determined by obtaining, from the medical records department, the inventory of patients who utilized this specialist clinic in a 12-month calendar year. An average of 250 new patients with schizophrenia was found to have utilized the facility in the past year. This constituted the sample frame.

The sample size of the present study was determined using the sample size estimation table for a given sample frame (20). According to the table, for a sample frame of 250, the minimum sample size required to be studied is 152, at 95% CI. Thus, a minimum sample of 152 was sought.

### Sample Selection

**Figure 1:**
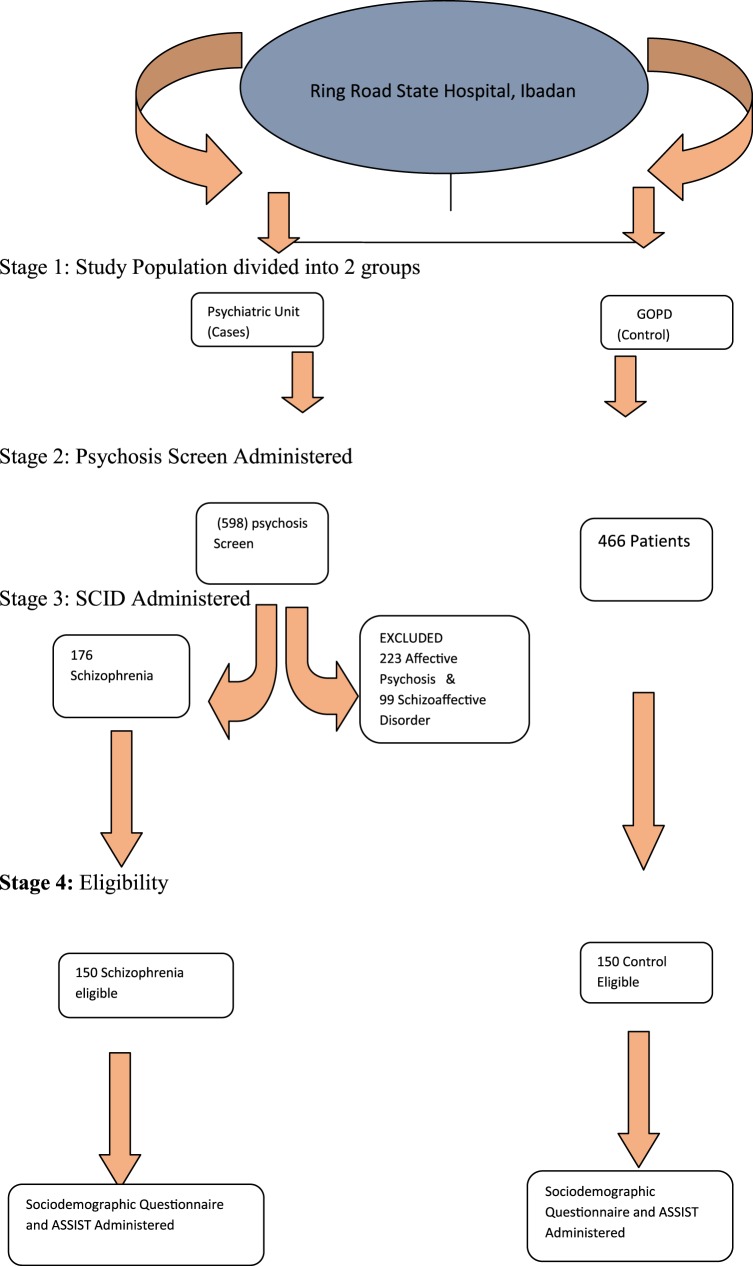
**Sampling technique**. Stage 1: study population divided into two groups. Stage 2: psychosis screen administered. Stage 3: SCID administered. Stage 4: eligibility.

Participants who met study criteria were consecutively recruited. All patients who utilized the psychiatric unit of the State Hospital, Ring Road, Ibadan, were initially screened with the psychosis screening questionnaire (21). Those who screened positive had the structured clinical interview for DSM-IV axis I disorder (SCID) administered to them. Those with diagnoses of schizophrenia proceeded to the second stage of the study, in which the Alcohol and Substance Involvement Screening Test (ASSIST) was administered to determine the prevalence of cannabis use and the risk of health problems, the procedure of sample selection is shown in Figure 1.

#### Inclusion Criteria

All participants were required to meet DSM-IV criteria for schizophrenia and must have been suffering from the illness for a period of at least 2 years. They were also expected to be accompanied by a principal caregiver with whom collateral information could be obtained.

#### Exclusion Criteria

1. Patients with schizoaffective disorder and affective psychosis.
2. Patients with any major general medical conditions.
3. Patients who were not accompanied by any principal caregiver.

##### Control Group

The control group consisted of patients who attended GOPD of the State Hospital, Ring Road, Ibadan, for simple ailments during the study period. The SCID was also administered to the control group.

#### Exclusion Criteria for the Control Group

1. Patients with any lifetime or current DSM-IV axis I disorder, according to the SCID.
2. Patients with a lifetime or current DSM-IV axis II disorder, according to the SCID.
3. Patients with a family history of any psychiatric illness were also excluded.

Patients in the control group who met eligibility criteria were matched by age and gender with those with schizophrenia. This was because of the effect of age and gender on the prevalence of drug use, according to previous literature (22).

##### Caregiver

For the purpose of this study, a caregiver was defined as “a non-professional person in the community who was most involved with the everyday care of the case and would be very likely to respond to any request for special assistance at any time, if such a request was made by the case” (23).

### Ethical Considerations

Permission for the study was obtained from the ethical and review board of the Ring Road State Hospital as directed by the department of planning, research, and statistics, the ministry of health Oyo state, to ascertain that the study met criteria for human research for experiments involving human beings.

Informed consent was obtained from each of the patients and or their relations, after the study was fully explained to them.

### Measures

#### Sociodemographic Questionnaire

Information about sociodemographic characteristics of respondents, including the age of respondents, gender, educational background, the age at the onset of illness, and duration of untreated psychosis was obtained.

#### The Psychosis Screening Questionnaire

The psychosis screening questionnaire (21) is a simple 6-item yes/no option instrument that probes into lifetime history of hypomania, thought interference, persecution, perceptual abnormalities, strange experiences, and hallucination. This short instrument is also a valid instrument for screening for psychosis and has been previously used in Nigeria (23).

#### World Mental Health Structural Clinical Interview for DSM-IV Axis 1 Disorder 2010 Version (SCID)

The SCID (24) was used to generate diagnoses of the subjects. The instrument covers all the criteria for the diagnoses included in the various modules. The interviewer makes a clinical judgment as to whether each criterion is met. The instrument is available in a patient edition for use with subjects who have been identified as psychiatric patients and in a non-patients edition suitable for use in epidemiological studies.

#### Positive and Negative Syndrome Scale

*Positive and negative syndrome scale* (PANSS) is a 30-item, 7-point rating instrument that has adapted 18 items from the Brief Psychiatric Rating Scale (BPRS) (25) and 12 items from the Psychopathology Rating Schedule (PRS) (26). The PANSS addresses both the presence and severity of symptoms, and the highest applicable rating point is always assigned, even if the patient meets criteria for lower ratings as well. Of the 30 psychiatric parameters assessed on the PANSS, 7 were chosen *a priori* to constitute a positive scale, 7 make up a negative scale and the remaining 16 general psychopathology.

#### The Alcohol, Smoking and Substance Involvement Screening Test

The ASSIST was used to obtain information about lifetime and current cannabis use.

The ASSIST is a cross-culturally validated instrument that was developed by the World Health Organization to screen for drugs, alcohol, and tobacco use in high prevalence settings and has been previously used in Nigeria (27).

The ASSIST-linked brief intervention is a short intervention lasting 3–15 min given to clients who have been administered the ASSIST by a health worker. The ASSIST screens for the use of all substance types (tobacco products, alcohol, cannabis, cocaine, amphetamine-type stimulants (ATS), sedatives, hallucinogens, inhalants, opioids, and “other” drugs) and determines a risk score (“lower,” “moderate,” or “high”) for each substance (28).

The risk scores are recorded on the ASSIST feedback report card, which is used to give personalized feedback to clients by presenting them with the scores that they have obtained and the associated health problems related to their level of risk. Asking clients if they are interested in viewing their scores allows the health worker to commence a discussion (brief intervention) with the client in a non-confrontational way.

Screening and brief intervention aim to identify current or potential problems with substance use and motivate those at risk to change their substance use behavior by creating a connection between their current pattern of use and the associated risks and harms (29). In general, brief interventions in primary care ranges from 3 min of brief feedback and advice to 15–30 min of brief counseling (30).

##### Scoring

For drugs such as cannabis, scores of 0–3 indicates lower risk, 4–26 moderate risk, and 27 and above, high risk.

The modality of the brief intervention is determined by the risk score. For clients scoring in the lower risk range (0–3), general health advice is given; for a moderate risk score (4–26), brief intervention and take-home booklet and information are prescribed; while for high-risk score (>26) brief intervention, take-home booklet, and information and referral to specialist hospital for assessment and treatment constitute the components of the intervention.

The outcome of the intervention given to the sample studied in the current study is beyond the scope of this report.

### Data Management and Analyses

The questionnaires were serialized, cleaned, edited, and safely stored. Thereafter, the information yielded by each respondent was entered directly into the computer using the SPSS software version 15.0 (SPSS, Inc., Chicago, IL, USA). Chi-square statistics were used to analyze categorical data for within-group comparisons of patients with schizophrenia. All Chi-Square were Yates-corrected for a 2 × 2 comparison or Bonferroni-corrected when there were multiple comparisons. Whenever a cell was <5 in any 2 × 2 table, Fisher’s exact test was used. The McNemar test was used to determine significant differences in two-level categorical variables between the cases and the control and conditional logistic analyses was used for multilevel categorical data. The Independent *t*-test was used to test for differences between the mean scores of the two groups. The association between cannabis use and PANSS score among patients with schizophrenia was determined using Pearson correlation. Since the response to cannabis use was bivariate and that of PANSS was multinomial, each of the PANSS subscales was dichotomized using the median score for carrying out the correlation analysis. A correlation analysis was also carried out between each of the subscales of the PANSS and the ASSIST. A Bonferroni correction was made for each of the *p*-value obtained by dividing the critical *p*-value of 0.05 with the number of comparisons made, i.e., 0.05/4, in the case of correlation analysis. All analyses were carried out within 95% CI, *p* < 0.05.

## Results

A significantly higher proportion of patients with schizophrenia were unmarried and unemployed compared with the control, *p* < 0.001. Prevalence of cannabis use among the cases and the control group was 10.0 and 2.7%, respectively. This was significant at *p* = 0.03. Mean ASSIST score was also significantly higher among the cases compared with the control, *t* = 6.2, *p* < 0.001. A significantly higher proportion of cases were at moderate health risk from cannabis use compared to those who were at low health risk, *p* = 0.02 (Table 1).

**Table 1:** **Sociodemographic characteristics of respondents**.

Variables	Cases *N* = 150		Control *N* = 150		Statistics	
	*N*	%	*N*	%	RR(CI)	*p*
**Age (years)**						
<25	46	30.7	42	28.0	1.06 (0.83–1.36)	0.70
25–34	54	36.0	44	29.3	1.16 (0.92–1.46)	0.26
35–44	30	20.0	34	29.7	0.92 (0.69–1.23)	0.67
>44	20	13.3	30	20.0	0.76 (0.54–1.10)	0.16
**Gender**						
Male	58	38.7	70	46.7	0.84 (0.67–1.07)	0.20
Female	92	61.3	80	53.3		
**Marital status**						
Married	68	45.3	124	82.7	0.46 (0.37–0.54)	<0.001
Unmarried	82	54.7	26	17.3		
**Employment status**						
Employed	76	50.7	142	94.7	0.38 (0.32–0.47)	<0.001
Unemployed	74	49.3	8	5.3		
**Education**						
No formal	8	5.3	10	6.7	0.88 (0.52–1.50)	0.81
Elementary	26	17.3	30	20.0	0.91 (0.67–1.24)	0.66
Secondary	72	48.0	64	42.7	1.11 (0.88–1.40)	0.42
Tertiary	44	29.3	46	30.7	0.95 (0.71–1.28)	0.88
**Occupation**						
High level professional	1	0.7	7	4.7	0.24 (0.04–1.54)	0.07
Skilled worker	15	10.0	80	53.3	0.24 (0.15–0.39)	<0.001
Semi-skilled/unskilled	60	40.0	55	36.7	1.07 (0.85–1.35)	0.64
Unemployed	74	49.3	8	5.3	2.59 (2.13–3.15)	<0.001
**Religion**						
Christianity	53	35.3	48	32.0	1.08 (0.85–1.36)	0.63
Islam	97	64.7	102	68.0		
**Ethnicity**						
Yoruba	90	60.0	85	56.6	1.07 (0.85–1.35)	0.64
Igbo	35	23.3	40	26.7	0.91 (0.69–1.20)	0.59
Hausa	15	10.0	12	8.0	1.12 (0.79–1.61)	0.69
Minority tribes	10	6.7	13	8.7	0.86 (0.53–1.39)	0.66
Current Cannabis use	15	10.0	4	2.7	1.64 (1.26–2.14)	0.02
Mean (SD)ASSIST Score	19.87	5.31	3.00	0.81	6.2^t^	<0.001
**ASSIST Score/Health Risks**						
0–3 (Low health risk)	1	25.0	3	75.0	5.2^X^	0.02^FE^
4–26 (Moderate health risk)	14	93.3	1	6.7		
>26 (Severe health risk)	–	–	–	–		

*FE, Fisher’s Exact p-value; t, Independent t-test; X, Chi square*.

Respondents who were of the male gender and those who were not married were significantly more likely to be cannabis users among patients with schizophrenia, *p* < 0.001 and *p* = 0.01, respectively (Table 2).

**Table 2:** **Sociodemographic characteristics of cannabis use among patients with schizophrenia (*N* = 150)**.

	Yes		No			
	*n*	%	*N*	%	χ^2^	*p*
**Age (years)**						
<25	–	–	16	100.0	4.8	0.2
25–34	7	15.9	37	84.1		
35–44	2	5.0	38	95.0		
>44	6	12.0	44	88.0		
**Gender**						
Male	13	22.4	45	77.6	15.7	<0.001^FE^
Female	2	2.2	90	97.8		
**Marital status**						
Married	2	2.9	66	97.1	5.2	0.01^FE^
Unmarried	13	15.9	69	84.1		
**Employment status**						
Employed	7	9.2	69	90.8	0.1	0.7
Unemployed	8	10.8	66	89.2		
**Education**						
No formal	–	–	8	100.0	3.3	0.07
Elementary	–	–	26	100.0		
Secondary	13	18.1	59	81.9		
Tertiary	2	8.7	42	95.5		
**Duration of illness**						
<3 years	6	7.9	70	92.1	0.8	0.4
≥3 years	9	12.2	65	87.8		
**Religion**						
Christianity	6	7.9	70	92.1	0.8	0.4
Islam	9	12.2	65	87.8		
**Ethnicity**						
Yoruba	10	11.0	80	89.0	0.7	0.8
Igbo	4	11.0	31	89.0		
Hausa	–	–	15	100.0		
Minority tribes	–	–	10	100.0		

*FE, Fisher’s exact*.

After Bonferroni correction, there was a significant correlation between cannabis use and Negative PANSS and also with total PANSS (*p* < 0.001 and *p* = 0.003), respectively (Table 3). Also, after Bonferroni correction, among patients with schizophrenia who were also current cannabis users, there was a significant correlation between ASSIST scores and negative PANSS on the one hand and ASSIST scores and general psychopathology subscale of the PANSS on the other hand. There was also a significant correlation between ASSIST scores and total PANSS score (*p* < 0.001) (Table 4).

**Table 3:** **Correlation between cannabis use and positive and negative symptoms scales in schizophrenia (*N* = 150)**.

		Cannabis use	Positive PANSS	Negative PANSS	General Psychopathology PANSS	Total PANSS
Cannabis	Pearson correlation	1	0.170^BNS^	0.298^BS^	0.183^BNS^	0.245^BS^
	Sig. (2-tailed)		0.039	0.000	0.026	0.003
Positive PANSS	Pearson correlation	0.170^BNS^	1	0.131	0.526^BS^	0.681^BS^
	Sig. (2-tailed)	0.039		0.109	0.000	0.000
Negative PANSS	Pearson correlation	0.298^BS^	0.131	1	0.645^BS^	0.752^BS^
	Sig. (2-tailed)	0.000	0.109		0.000	0.000
General psychopathology PANSS	Pearson correlation	0.183^BNS^	0.560^BS^	0.645^BS^	1	0.935^BS^
	Sig. (2-tailed)	0.025	0.000	0.000		0.000
Total PANSS	Pearson correlation	0.245^BS^	0.681^BS^	0.752^BS^	0.935^BS^	1
	Sig. (2-tailed)	0.002	0.000	0.000	0.000	

*BS, Bonferonni significant; BNS, Bonferonni not significant*.

**Table 4:** **Correlation between ASSIST scores and positive and negative symptoms scale in schizophrenia (*N* = 15)**.

		Correlations				
		Positive PANSS	Negative PANSS	General psychopathology PANSS	Total PANSS	ASSIST
Positive PANSS	Pearson correlation	1	0.332^BS^	0.609^BS^	0.724^BS^	0.512^BNS^
	Sig. (2-tailed)		0.009	0.006	0.000	0.025
Negative PANSS	Pearson correlation	0.332^BS^	1	0.651^BS^	0.719^BS^	0.730^BS^
	Sig. (2-tailed)	0.009		0.003	0.001	0.000
General psychopathology PANSS	Pearson correlation	0.609^BS^	0.651^BS^	1	0.950^BS^	0.823^BS^
	Sig. (2-tailed)	0.006	0.003		0.000	0.000
Total PANSS	Pearson correlation	0.724^BS^	0.719^BS^	0.950^BS^	1	0.844^BS^
	Sig. (2-tailed)	0.000	0.001	0.000		0.000
ASSIST	Pearson correlation	0.512^BNS^	0.730^BS^	0.823^BS^	0.844^BS^	1
	Sig. (2-tailed)	0.025	0.000	0.000	0.000	

*BS, Bonferonni significant; BNS, Bonferonni not significant*.

## Discussion

In this case–control study, 150 patients with schizophrenia were compared with an equal number of controls. Prevalence and determinants of cannabis use and associated health risk based on the ASSIST score were sought. Prevalence of cannabis use was 10.0% among the cases and 2.7% in the control group. Cannabis-related health risk as determined by the ASSIST score was significantly higher among patients with schizophrenia. Male gender and presence of psychopathology were found to be significant predictors of cannabis-related health risk.

To the best of the author’s knowledge, this is the first study on this subject matter in Nigeria. Thus, comparisons of the findings from the present study will be based on research from other parts of the world.

### Sociodemographic Characteristics of Patients

The sample of patients with schizophrenia was generally characterized by relatively young age compared with the controls. The mean age of the sample with schizophrenia was 34.3 ± 11.6 years. This was lower than the 38.7 ± 11.4 years obtained by Gureje and colleagues in 2002 (31). This finding is in line with the report of epidemiological catchment study, indicating that schizophrenia is commoner in the younger age group (32). In the present study, a higher proportion of the patients with schizophrenia were unmarried and unemployed, compared with the control group. Although it is difficult to determine which one preceded the other between being unmarried and psychoses, it has been found that patients with schizophrenia often live alone, were unmarried (33), and were unemployed (34). We found that male gender and being unmarried were associated with cannabis use among subjects with schizophrenia. This is in support of previous studies in this environment (22, 35). Remarkably, substance abuse tends to share certain sociodemographic characteristics like being unmarried and being unemployed with schizophrenia.

### Prevalence of Cannabis Use

In the present study, 10.0% of patients with schizophrenia were current cannabis users. This figure is within the reported range of 8.6–28.6% from a meta-analysis involving 10 studies carried out about 5 years ago (36). Our finding also lends support to previous evidence, indicating an association between adolescent cannabis use and the development of schizophrenia (1, 2–3). Although a direct cause and effect relationship was not proven, our finding that cannabis use was significantly associated with negative PANSS score may support the self-medication hypothesis; the argument being that cannabis is used as an adjunct by patients to attenuate their negative symptoms (8). On the other hand, the observed significant correlation between cannabis use and total PANSS score may suggest that cannabis use in schizophrenia is related to the presence of psychotic symptoms in the entire psychopathological process. Indeed, Kuepper and colleagues conceived cannabis use as impacting the symptoms of schizophrenia (37). Nevertheless, a common neurobiological pathway may be considered for this phenomenon, given a number of commonalities between schizophrenia and cannabis use (38, 39).

### Cannabis Use and Health Risks among Cannabis Users

We also found that among patients living with schizophrenia, who were cannabis users, only 1 was at low health risk and the remaining 14 were at moderate health risk. The findings have a lot of implications regarding the negative effects of chronic cannabis use. This is pertinent considering that cannabis consumption carries specific risks of dependence (40), lung cancer, tuberculosis, other lung diseases (41), and cardiovascular diseases (40).

Notable is the association between ASSIST scores and negative PANSS score, ASSIST scores and general psychopathology subscale of PANSS scale, as well as ASSIST scores and total PANSS score. This, by implication, suggests that the more severe the symptoms of schizophrenia the more grave the health risks of cannabis smoking. It is conceivable that individuals suffering from schizophrenia are not likely to seek treatment for their cannabis use or that they have difficulties adhering to a substance abuse treatment program. They are also more likely to have continued cannabis use, leading to clinical exacerbation, non-compliance with antipsychotic medications, and poor overall health.

The finding that 2.7% of service users in a walk-in clinic used cannabis is also an important epidemiological finding. Although a higher rate, about fourfold, was reported in a non-psychotic population over 10 years ago (42); this finding is notable considering the potential health risks associated with cannabis smoking. More so, none of them had ever sought treatment for their cannabis use.

### Implication of the Study

The results of the present study have implications for cannabis use screening in patients with schizophrenia. This is because of the complex association between cannabis use and schizophrenia. Great consideration for simultaneous implementation of a cannabis cessation program during treatment for schizophrenia is required, given the associated health risks.

The current study has a number of limitations. The prevalence of cannabis use could have been masked by significant under-reporting, given the sensitive nature of the question. Obtaining information on substance use without a face-to-face contact has been long recognized in improving response rate (43). The clinical state of the patient could also have led to difficulty in obtaining information about cannabis use, if the caregiver was not aware of such. Also, toxicological screen was not carried out, because of the economic implication of the study.

Our findings should be interpreted with caution, given the small sample size. This also limits the generalization of the research findings. However, an important strength of this study was the detection of a subsample of patients with schizophrenia and the control group who benefited from screening and brief intervention for cannabis use.

In conclusion, cannabis use is fourfold more prevalent in patients with schizophrenia. About 1 in every 40 persons in a *de facto* primary care, in this instance, the general outpatients’ department, a walk-in clinic use cannabis. Cannabis use in patients with schizophrenia is associated with moderate health risk. Although the present study has not established a causal relationship between cannabis use and schizophrenia, it should serve as a template for large-sample size prospective studies in Nigeria. Such study design may shed more light on the relationship between cannabis use and symptoms of schizophrenia.

## Author Contributions

VL conceived the idea and was responsible for study design, analysis, and manuscript writing. OA was responsible for data collection and was also involved in manuscript writing. Both authors gave a substantial contribution to the study.

## Conflict of Interest Statement

The authors declare that the research was conducted in the absence of any commercial or financial relationships that could be construed as a potential conflict of interest.
